# Flowers, Ferns, and Ward Decorations

**Published:** 1886-11-06

**Authors:** 


### FLOWERS, FERNS, AND WARD DECORATIONS.

[Continued from page 65.—Communications for this column should be addressed "Gardener," care of the Editor of THE HOSPITAL.]

"Is it nothing to you all ye who pass by?" AND now, my reader, should you have followed me thus far, there is one thing I would say, one thing in which I would ask and claim your aid, and it is this, that in some way or other you would try and bring together these treasures of horticulture—"earth's stars," as they have been called—and those poor brethren of our overcrowded cities [illegible] know them not, to whom the sights and sounds of the garden are as a dream—a dead language! There are thousands of human beings in this England of ours who do not know one flower from another; and we who have gardens, and who love their denizens (we do love them with a passion beyond many other loves and desires, outlasting the heat and burden of the days of small things), must realise the emptiness of lives like these and the blank such ignorance represents, with a fervent wish and determination to change, as far as in us lies, this woful vacancy into a greater knowledge of those gifts, that keep and make the world so fair a resting-place. There is no better or easier way of doing this than by helping to adorn the wards of our hospitals, workhouses, and infirmaries with plants and cut blossoms, and by doing this systematically throughout the year.

Giving Flowers. There are those lying there in the grip of disease, in the agony of pain, to whose eyes the sight of flowers may be as oases in a dreary desert, a break, a relief through hours of bitter endurance. "Somebody's darling" stretched on a narrow bed, in grievous pain, stricken in mind or limb, may rest his weary gaze on branch or blossom, and call God's blessing upon the giver's head, since it comes as a beloved memory, a breath of the outside brightness and life across the dreary monotony of his present surroundings. How many of those who hurry daily past the doors of our great hospitals pause to give a thought to those lying within, ever dream of doing anything to vary the sadness of the lot of those on whom it hath pleased their Creator to lay His hand in heaviness? "Is it nothing to you, all you who pass by?" Is there nothing you can do, who have health and strength for your portion, to alleviate the misery hidden away behind the walls, where the sick and dying of the human race await their passport into renewed vigour or the grave?

What all can do. Not much that many can do, certainly, but there is always this; everyone can send and bring flowers to the hospitals to cheer the sadness of Christ's little ones. "For as much as ye have done it unto the least of these, ye have done it unto Me," He has said. The rooms can be brightened—the rooms where pain and suffering are the chief visitors; the tables can be covered, the walls decorated, and the window-sill filled with green and fragrant things to enrich the air and break the dull severity inseparable from such places as these. Eyes that have met death face to face, struggled, fought with, and conquered him, may be greeted on their return to consciousness, their renewal of earth's penalties, by something fair and hopeful, something to make the ward where they lie less unhomelike. Those who have entered the hospital-doors to pass out never again in life, may have their journey to the Great Unknown made easier, if so be that their earthly glance fall, lastly, on something like a gentle herald of that unutterable good, awaiting God's poor, "where beyond these voices there is peace." Next week I propose to speak more at length on the subject of ward decoration, and the best and easiest method of carrying out the plan I have suggested.

